# Enhancing food security through reducing Champa rice losses: Assessing the impact of crop moisture and harvesting methods on yield quantity and quality

**DOI:** 10.1371/journal.pone.0326846

**Published:** 2025-07-09

**Authors:** Jamshid Kyani, Mahmoud Ghasemi-Nejad-Raeini, Afshin Marzban

**Affiliations:** Department of Agricultural Machinery and Mechanization Engineering, Faculty of Agricultural Engineering and Rural Development, Agricultural Sciences and Natural Resources University of Khuzestan, Mollasani, Iran; University of Minnesota, UNITED STATES OF AMERICA

## Abstract

Rice is a major staple crop globally, and minimizing harvest-stage losses is essential for improving production efficiency and food security. Among the critical factors in direct combine harvesting, the timing—determined by grain moisture content plays a crucial role in both yield quality and quantity. This study investigated the impact of harvesting methods and grain moisture levels on the quantitative and qualitative losses of Champa rice in Ramhormoz County during the 2019 growing season. A factorial experiment was conducted using a Randomized Complete Block Design (RCBD) with three replications. The factors included three grain moisture levels (13–15%, 23–25%, and 39–41%) and three harvesting methods (manual, rice combine, and grain combine). Results indicated that at low moisture levels, the highest paddy loss (9.53%) occurred with manual harvesting, while the rice combine recorded the lowest loss (2.5%). Conversely, at high moisture levels, grain combine harvesting led to the highest grain breakage (25.66%) and cracked grains (30.66%), whereas manual harvesting resulted in the lowest cracked grain percentage (2.66%). The highest Head Rice Yield (HRY) (83.66%) was achieved at low moisture levels, with manual harvesting yielding the highest average HRY (83.11%). Interaction effects revealed that the combination of manual harvesting and low moisture content produced the highest HRY (86%), while grain combine harvesting at high moisture resulted in the greatest proportion of broken rice (33.66%). These findings highlight the significance of selecting appropriate harvesting methods and timing to reduce losses and enhance rice quality. The type of combine also significantly influenced the mechanical stress on grains. The study recommends using rice combines at lower moisture levels to minimize losses and avoiding grain combine usage under high moisture conditions. Optimizing harvesting practices not only improves rice processing efficiency but also contributes to food security by maximizing yield quality without the need for land expansion.

## 1. Introduction

Agriculture, as one of the fundamental pillars of the global economy, plays a crucial role in ensuring food security and economic sustainability. Rice (Oryza sativa), as one of the most important staple crops, serves as the primary food source for more than half of the world’s population. With increasing global demand for food and limited arable land, enhancing productivity and minimizing post-harvest losses are essential for sustainable development.

Post-harvest losses in rice, especially during harvesting, represent a major challenge in the supply chain of this essential crop [1–3]. These losses can be classified into chemical and physical categories. Chemical losses arise from factors such as elevated moisture content and product spoilage, while physical losses, which can be either visible or hidden, occur due to mechanical impacts during threshing and transportation [4,5].

Numerous studies have indicated that various factors, including grain moisture, temperature, cultivar type, and crop condition, significantly influence harvest losses [6,7]. For instance, the grain’s moisture content at harvest plays a pivotal role in reducing grain breakage and enhancing milling recovery [8].

Mechanical factors such as the clearance between threshing cylinders and grain moisture also affect the final product quality [9]. Alwan-Alsharifi et al. (2017) [10] found that a clearance of 0.8 mm and a grain moisture range of 10–12% produced the best results in minimizing broken grains and maximizing hulling efficiency [10]. Moreover, the type of whitening machine and grain moisture during processing significantly impact the percentage of broken and cracked grains [11–14]. For example, abrasive-type whitening machines operating at a grain moisture level of 8–9% have been reported to yield the lowest percentage of broken rice.

Post-harvest drying conditions and final moisture content are also crucial factors. Generally the drying temperatures of 40–45°C and a final moisture content of 12–13% led to a reduction in grain cracking [15]. Additionally, research has shown that timely harvesting, typically when grain moisture ranges between 18% and 20%, maximizes milling yield and minimizes grain breakage [16–19].

The effective management of the rice production continuumincluding pre-harvest, harvest, and post-harvest stages is essential for maintaining grain quality and minimizing losses. This includes appropriate harvest timing, drying methods, machinery settings, and processing techniques. These practices not only enhance the economic value of the crop but also improve farmers’ livelihoods and contribute to reducing food waste [20–23].

Globally, the necessity of reducing food losses extends beyond regional concerns. With the rapid increase in the world population and the urgent need for food security, addressing rice production losses can have a direct impact on global sustainability. It is estimated that nearly one-third of the world’s edible food (1.3 billion metric tons) is wasted annually [2,24–26], leading not only to food insecurity but also to the depletion of natural resources and environmental degradation.

Preventing rice losses and improving its quality will not only enhance productivity and improve farmers’ livelihoods but also contribute to global sustainability efforts [27]. Reducing waste means optimizing the use of limited resources, protecting the environment, and ensuring higher-quality food [28]. In this regard, modern and mechanized harvesting techniques can serve as effective tools in achieving these objectives.

Although extensive research has examined the roles of grain moisture and quality attributes in rice, there remains a lack of studies addressing the interactive effects of harvesting techniques and moisture levels under the specific agro-climatic conditions prevalent in southern Iran, particularly its high temperature and humidity. This gap is notably significant for the Champa rice variety, which continues to dominate cultivation in the region due to its desirable consumer qualities and agronomic performance. Therefore, the present study aims to bridge this knowledge gap by investigating how different harvesting methods influence both quantitative and qualitative losses in Champa rice cultivated in Ramhormoz one of the key rice-growing regions in Khuzestan Province. By addressing this localized challenge within a broader context of food security and resource efficiency, the study offers practical insights for enhancing grain quality and reducing post-harvest losses. Ultimately, its findings are expected to support more resilient rice production systems while contributing to national and global efforts toward sustainable agriculture.

## 2. Materials and methods

### 2.1. Study area and experimental period

This study was carried out in the fall of 2019 to evaluate the impact of various mechanized harvesting methods on both quantitative and qualitative losses in the Champa rice variety. The experiment took place in Ramhormoz, a major rice-producing region situated 89 km west of Ahvaz, Iran. The study area is located at 31°16’ N latitude and 49°37’ E longitude, at an elevation of 160 meters above sea level.

According to long-term meteorological records, Ramhormoz experiences a warm and semi-arid climate, with an average annual rainfall of approximately 390 mm. The region’s mean annual temperature is around 30°C, with recorded extremes ranging from a minimum of 10°C to a maximum of 53°C.

### 2.2. Experimental procedure

A uniform 10-hectare field was selected for this study. To maintain consistent conditions throughout the experiment, three plots with identical sowing density, planting date, and agronomic management were designated. Harvesting was conducted at three different times to achieve the targeted grain moisture levels required for the study.

To estimate field yield, each experimental plot was divided into three equal sections. From each section, a 1-square-meter sample was manually harvested using a sickle to avoid grain shattering. The grains were then manually separated, and yield was calculated for each sample.

To assess natural pre-harvest losses, prior to harvesting, fallen grains and panicles on the soil surface were collected from each plot and stored in separate bags. Following this, the combine harvesters were properly calibrated, and harvesting was carried out under uniform conditions. The harvesting locations within each plot and for each treatment were selected randomly. Additionally, all combine settings were adjusted by a trained specialist before harvesting.

In this study, two independent variables were purposefully selected based on initial field observations and expert consultation. These included the real-time grain moisture at harvest (measured using a precision moisture meter) and the harvesting method (based on dominant regional practices), both identified as the most critical and controllable factors influencing harvest losses in the target region.

The experimental design was structured to isolate the effects of these two dominant factors while minimizing the influence of potential confounding variables, given their direct connection with harvesting practices and post-harvest management in the region.

Although factors such as operator skill, soil condition, and machine age can also affect harvest losses, efforts were made to control and stabilize these variables within the experimental setup to reduce their impact on the results.

### 2.3. Experimental design

The experiment was structured as a factorial arrangement within a randomized complete block design (RCBD) with three replications (Table 1), ensuring statistical reliability while accounting for field variability [29].

**Table 1:** Treatments used in field experiments.

Treatments	Treatment levels
Harvest moisture	(1) Harvest moisture (13–15%), (2) Harvest moisture (23–25%) and (3) Harvest moisture (39–41%)
Harvest methods	(1) Manual harvesting (2), Rice combine harvesting and (3) Grain Combine Harvesting

The independent factors evaluated in this study were:

1. Harvesting moisture content
2. Harvesting method

The dependent variables assessed were:

(a)  Quantitative losses
(b)  Qualitative losses of rice grains

### 2.4. Specifications of the combines used in the study

In this study, two combines were used: the John Deere 1055 grain combine, manufactured by Iran Combine Manufacturing Company and equipped with a spike-tooth threshing system, and the K-BOS (Suzuki) 4LZ-3.0 rice combine, manufactured in China. Some of the technical specifications of these two combines are presented in Table 2.

**Table 2:** Technical specifications of combines used.

Technical specifications	Grain combine 1055	Rice combine K-BOS
Cutting width (m)	3.03	2.30
Threshing type	Pin tooth thresher	Dual thresher-equipped with dual steel thresher shaft
Tank capacity (liters)	2700	1600

### 2.5. Measurement of harvesting moisture content

To determine the moisture content of paddy rice, a digital moisture meter (Model: HT-PRO8300021, manufactured by Farmex) was employed at three different moisture levels. The device operates within a moisture range of 8% to 45%, with an accuracy of ±0.7%, and a working temperature range of 0–49°C.

### 2.6. Measurement of quantitative losses

To evaluate harvesting losses, measurements were performed according to the ISIRI 6133 standard [30]. Losses were classified based on the functional units of the combine harvester as follows: natural losses, cutting unit losses, threshing and separation losses, and cleaning unit losses [31,32]. Experiments were conducted in three replications using a randomized block design to minimize environmental effects and enhance result accuracy.

#### 2.6.1. Natural losses.

These losses refer to grain shedding before harvesting. To quantify them, random sampling frames (0.5 × 0.5 m) were placed in the field before the combine passed. All grains found within the quadrats were collected and weighed to determine the percentage of natural losses relative to the total yield.

#### 2.6.2. Cutting unit losses.

These losses occur at the header section due to improper cutting or shattering. After the combine passed, sampling frames were placed just behind the cutter bar area, and all uncollected or shattered grains were gathered. The samples were cleaned and weighed to determine cutting losses as a percentage of total yield.

#### 2.6.3. Threshing and separation unit losses.

This category includes grains not separated properly by the threshing cylinder and those lost through the straw walkers. Sampling frames were placed behind the straw outlet of the combine, and grains present in the discharged straw were manually separated, weighed, and expressed as a percentage of the total yield.

#### 2.6.4. Cleaning unit losses.

These losses result from improper separation by the sieves and fan system. After harvesting, samples were collected from the residual lightweight materials and chaff discharged from the rear of the combine. Grains embedded in the light straw were separated, cleaned, and weighed to calculate cleaning unit losses.

#### 2.6.5. Total harvesting losses.

Total losses were calculated as the sum of losses from all the aforementioned units:

1. Natural losses
2. Cutting unit losses
3. Threshing and separation unit losses
4. Cleaning unit losses

### 2.7. Measurement method for percentage of paddy breakage and cracking

To accurately assess the effect of different treatments on the extent of breakage and cracking in paddy grains prior to milling, a total of 100 paddy grains were randomly selected. These grains were carefully dehulled manually to avoid any mechanical stress and were then examined using a crack detection device. By illuminating the grains from beneath, cracked or broken grains were identified. The results were recorded as percentages relative to the total number of grains. The percentage of broken paddy grains (Breakage of Paddy (%)) and cracked grains (Cracked Paddy (%)) were calculated separately and considered as key indicators in the evaluation of the physical quality of the paddy. This method was conducted in accordance with the approaches outlined by Aquerreta et al. (2007) and Latifi (2014) [33,34].

### 2.8. Qualitative assessment of milled rice

The qualitative assessment of milled rice encompasses several critical indicators that collectively determine its market value, consumer acceptability, and overall processing quality. These indicators include Milling Recovery (MR), Milling Degree (MD), Head Rice Yield (HRY), Broken Rice Percentage (BR %), and Cracked Rice Percentage (CRP %) [34–36]. Each parameter reflects a specific aspect of rice quality from the efficiency of the milling process to the physical integrity of the grains thus offering a comprehensive understanding of the rice’s suitability for commercial and domestic purposes.

#### 2.8.1. Milling recovery (MR).

Milling Recovery represents the percentage of polished white rice obtained from the initial weight of paddy, serving as a key indicator of milling efficiency. It is calculated by dividing the total weight of the milled white rice by the original weight of the paddy and then multiplying the result by 100 to express it as a percentage. In this study, Milling Recovery was calculated using [35]:

Equation 1:


$$MR=\frac{Weight\;of\;White\;Rice\;(g)}{Weight\;of\;Paddy\;Rice\;(g)}\;\times 100$$ (1)

#### 2.8.2 Milling degree (MD).

Milling Degree indicates the extent to which the bran layer has been removed from the grain during the milling process. It directly influences the whiteness, texture, and nutritional content of the rice. This parameter is calculated as the ratio of the weight of white rice to the weight of husked (brown) rice, multiplied by 100. In this study, MD was determined using [36]:


$$MD=\frac{Weight\;of\;White\;Rice\;(g)}{Weight\;of\;Husked\;Rice\;(g)}\;\times 100$$ (2)

#### 2.8.3 Head rice yield (HRY) and broken rice percentage (BR %).

Head Rice Yield (HRY) refers to the percentage of whole, unbroken milled rice kernels (defined as having a length ≥ ¾ of the original paddy kernel) in relation to the total milled rice weight. A higher HRY value is indicative of minimal mechanical damage during harvesting and processing, reflecting superior grain quality and economic value. In parallel, Broken Rice Percentage (BR %) denotes the proportion of fragmented grains, which diminishes both the market value and cooking quality. In this study, a 20 g representative sample was used, and rice kernels shorter than ¾ of the original length were classified as broken. Both HRY and BR% were calculated using [34]:


$$Broken\;\;rice\left(\%\right)=\left(\frac{w_{b}}{w_{t}}\right)\times 100$$ (3)

Where:

$w_{b}$: Weight of broken rice (g)

$w_{t}$: Total weight of the milled rice sample (g)

Subsequently, the Head Rice Yield was determined by subtracting the broken rice percentage from 100, as shown:


Headriceyield(%)=100−Brokenrice(%) (4)

### 2.9. Statistical analysis

The experiment was conducted using a randomized complete block design (RCBD) in a full factorial arrangement with three replications, allowing for accurate assessment of treatment effects while accounting for variability across blocks. All data were analyzed using SAS statistical software, and treatment means were compared using the least significant difference (LSD) test at a 5% significance level.

Graphical representations of the results were created in Microsoft Excel to visually illustrate treatment effects and interactions. The statistical model used for the analysis is presented in [29]:


$$Y_{ijk}=\mu +R_{k}+A_{i}+B_{j}+{\left(AB\right)}_{ij}+\varepsilon_{ijk}$$ (5)

Where:

$Y_{ijk}$ = Observed value for the i-th level of factor A, j-th level of factor B, and k-th block (replication)

μ = Overall mean

$R_{k}$ = Effect of the kth replication (block)

$A_{i}$ = Effect of the ith level of factor A

$B_{j}$ = Effect of the jth level of factor B

${\left(AB\right)}_{ij}$ = Interaction effect between factors A and B

$\varepsilon_{ijk}$ = Random error associated with each observation

## 3. Results and discussion

### 3.1. Evaluation of rice harvest losses

#### 3.1.1. Impact of harvest moisture content and harvesting method on paddy losses.

Table 3 shows a statistically significant effect (P < 0.01) of both harvest moisture content and harvesting method, as well as their interaction, on total losses, broken grains, and cracked grains.

**Table 3:** Analysis of variance for the effect of harvest moisture content and harvesting method on paddy traits.

Source of variation	df	Mean squares		
		Losses (%)	Breakage of paddy (%)	Cracked paddy (%)
Replication	2	0.0023	5.48	1.037
Harvest moisture content	2	1.43**	169.04**	274.70**
Harvesting method	2	45.89**	297.93**	476.70**
Harvest moisture content × Harvesting method	4	7.94**	104.6**	94.37**
Error	16	1.6	2.89	2.26
CV	–	7.87	25	15.47

**: Significant at the 1% level,*: Significant at the 5% level and ^ns^: not significant.

The analysis of variance confirms that all three parameters were significantly influenced by these factors, highlighting their critical role in determining rice quality during harvest.

**Fig 1:**
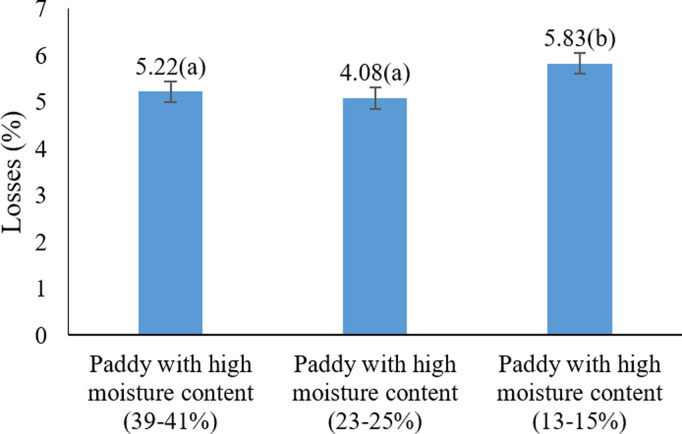
Effects of harvest moisture on paddy losses under different harvesting methods * Means with similar letters on each chart do not have a statistically significant difference (LSD).

As shown in Fig 1, the highest percentage of rice losses occurred at a moisture content of 39–41%, while the lowest losses were observed in the 13–15% and 23–25% moisture groups, which were not significantly different.

These findings indicate that higher moisture contents lead to more fragile paddy grains, making them more prone to damage and loss during harvesting.

This observation is consistent with previous reports showing a direct relationship between increased harvest moisture and higher grain loss, as moisture makes grains more vulnerable to mechanical impacts [37–39].

In support of these findings, Sharifi Sangdeh (2017) [40] reported that decreasing grain moisture from 22% to 19% led to a 68% reduction in losses, while increasing it from 17% to 19% resulted in a 65% reduction [40].

Similarly, Wang et al. (2024) [41] and Safari, Alizadeh, & Gerami, (2014) [42] found the lowest loss rate at a moisture content of 17.06%, emphasizing the importance of harvesting within this optimal range.

**Fig 2:**
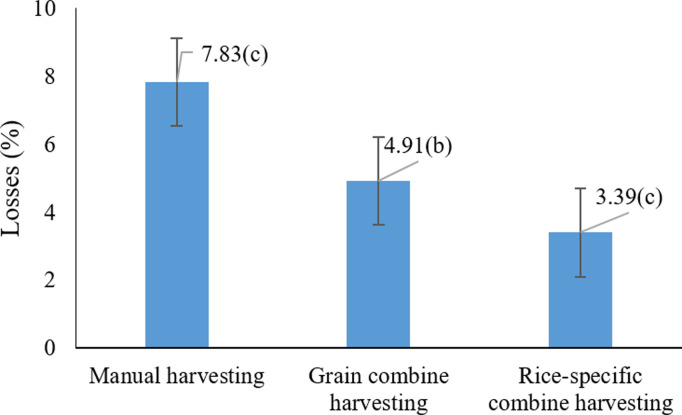
Effects of harvesting method on paddy losses * Means with similar letters on each chart do not have a statistically significant difference (LSD).

The results indicate that both harvesting method and grain moisture content significantly influence rice quality and grain loss. Fig 2 illustrates that among the three harvesting methods, manual harvesting led to the highest losses, peaking at 7.8% (P < 0.05), likely due to increased mechanical impact and vibration from the sickle, which caused greater dislodgement and grain loss. In contrast, combine harvesters particularly those equipped with dual blades demonstrated substantially lower losses. These findings are consistent with earlier studies emphasizing the role of harvesting method in determining post-harvest losses. For example, Sharifi Sangdeh (2017) [40] reported that grain combine harvesters reduced losses to 2.395%, compared to 1.221% for manual methods, suggesting the superior efficiency of machinery specifically designed for rice harvesting [40]. Similarly, Rahmati et al. (2014) [43] observed that rice-specific combines achieved a loss rate of only 2.32% in the Shirvan Chardaval region, significantly outperforming conventional grain combines (3.816%) [43]. Additionally, high moisture content at the time of harvest was found to reduce the mechanical strength of grains and increase the percentage of cracked kernels, as reported by Ilieva et al. (2025) [44] and Alwan-Alsharifi et al. (2017) [10]. The current study supports these findings by showing a statistically significant increase in cracked grains under high-moisture conditions. Mechanical damage during harvesting is exacerbated when using cereal combines at high grain moisture levels, highlighting the need for precise timing and machine calibration. Therefore, it is recommended that both the harvesting method and moisture conditions be carefully managed to minimize grain loss and maintain product quality.

**Fig 3:**
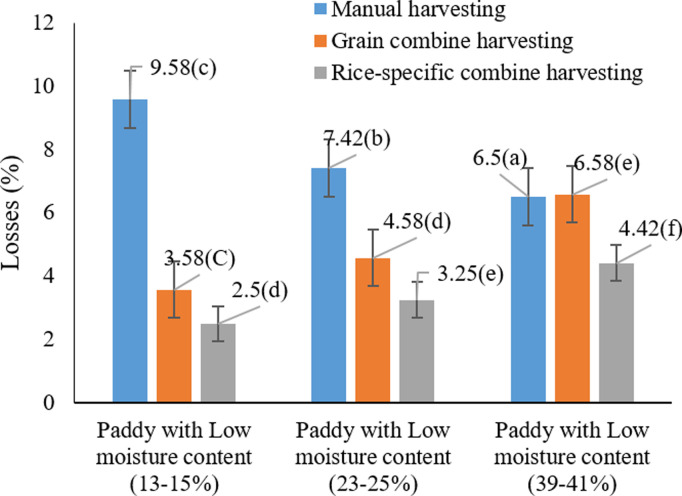
Interaction effects of harvest moisture and harvesting method on paddy losses* Means with similar letters on each chart do not have a statistically significant difference (LSD).

Fig 3 illustrates that the highest grain loss (9.58%) occurred under two specific conditions: manual harvesting at both low (13–15%) and high (39–41%) moisture contents. This pattern suggests that manual harvesting consistently results in higher losses, while extreme moisture levels further intensify these effects. The interaction between moisture content and harvesting method appears to play a critical role in determining overall losses. These results align with prior research emphasizing the importance of optimizing both harvest timing and method. For instance, Sharifi Sangdeh (2017) [40] and Zareiforoush, Komarizadeh, and Alizadeh (2009) [45] and Adewoyin (2023a) [46] confirmed that appropriate moisture management can significantly mitigate grain losses. Furthermore, Firouzi and Alizadeh (2014) [47] demonstrated that harvesting Hashemi rice 30 days after 50% flowering, when moisture reached 8.5% on a dry basis, led to improved milling efficiency and reduced breakage. Collectively, these findings underscore the necessity of synchronizing harvesting operations with optimal moisture conditions to reduce losses and enhance grain quality.

Consistent with the results in Fig 3, Sharifi Sangdeh (2017) [40] and Chandrajitha et al. (2016) [48] reported a significant reduction in grain losses as harvest moisture content declined from 22% to 19%, and from 17% to 19%, respectively. These findings highlight the critical role of precise moisture control in minimizing losses. Additionally, comparison of different combine harvesters showed no statistically significant difference (P ≥ 0.01), although the 4LZ-2.5A model had the lowest recorded loss (1.32%) and the ICR20 the highest (1.41%), indicating that rice-specific combines are generally more efficient [49]. Interestingly, Khanpour-Lehi et al. (2012) [49] observed an opposite trend: losses decreased as moisture content increased from 20% to 30%, with minimum loss at 30% (25%) and maximum at 20% (52%). Although this deviation may be attributed to differences in equipment and field conditions, it still supports the broader conclusion that harvester design particularly features like dual-deck cleaning systems and adjustable airflow plays a crucial role in reducing post-harvest losses during the cleaning phase.

#### 3.1.2. Effect of harvesting moisture and harvesting method on paddy cracking.

**Fig 4:**
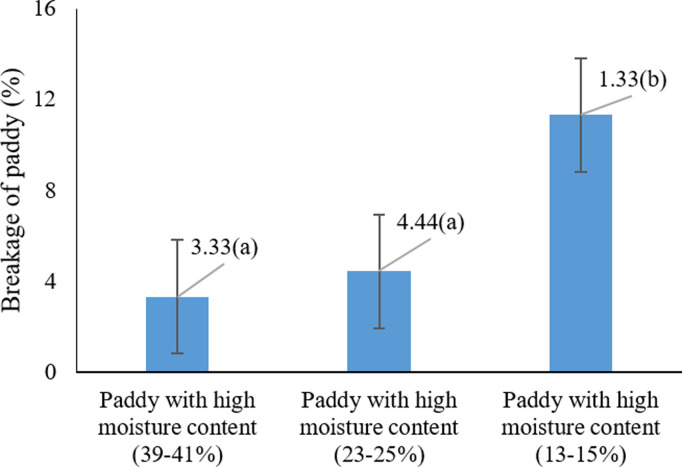
Effects of different harvest moistures on paddy cracking * Means with similar letters on each chart do not have a statistically significant difference (LSD).

The analysis of variance results (Table 3) showed that the effects of harvesting moisture, harvesting method, and their interaction on rice grain breakage were significant (P < 0.05). Fig 4 illustrates the comparison of different harvesting moisture levels on grain breakage. According to this chart, the highest rice breakage (11.33%) was observed at a harvesting moisture of 39–41%. No significant difference was found between the moisture levels of 13–15% and 23–25%, and both were statistically grouped together (P > 0.05). It appears that at higher moisture levels, rice grains lose firmness, resulting in increased breakage.

Previous studies confirm that high moisture content at harvest can increase grain breakage [50]. For example, one study reported that increasing rice moisture at harvest decreased both milling recovery and the percentage of whole grains [51]. Additionally, Firouzi, & Alizadeh (2014) [47] found that harvest timing and rice moisture content directly affected the milling recovery of whole grains, indicating that harvesting at the optimal time and moisture level can reduce grain breakage. In conclusion, harvesting rice at elevated moisture levels results in greater grain breakage. Therefore, optimizing harvest timing to ensure appropriate grain moisture is recommended to minimize losses and maintain high product quality.

**Fig 5:**
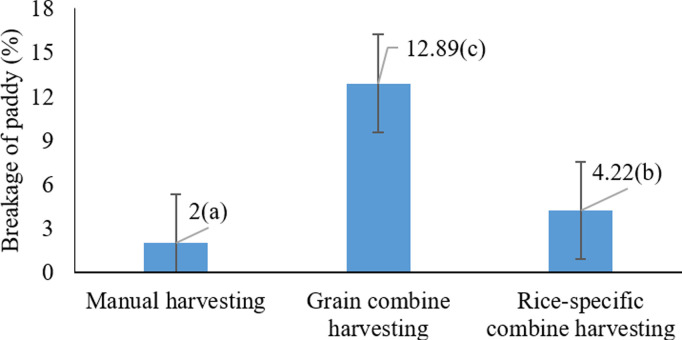
Effects of harvesting method on paddy cracking * Means with similar letters on each chart do not have a statistically significant difference (LSD).

As shown in Fig 5, the highest rice grain breakage (12.89%) occurred when using a cereal combine harvester. These results suggest that cereal combine harvesters, due to their design and operational mechanism, impart more mechanical stress on the grains, thereby increasing the rate of breakage. In contrast, rice-specific combine harvestersdesigned specifically for rice harvesting cause less damage to the grains and result in lower rice breakage. Similar observations have been reported in earlier studies, indicating that cereal combine harvesters typically result in higher grain damage and a greater percentage of breakage [21,46].

According to Kianni (2023) [52], grain breakage at harvest significantly affects the final product’s quality and yield. Under high grain moisture conditions, the likelihood of breakage increases. Thus, the proper design and adjustment of combine harvesters can play a critical role in reducing damage.

**Fig 6:**
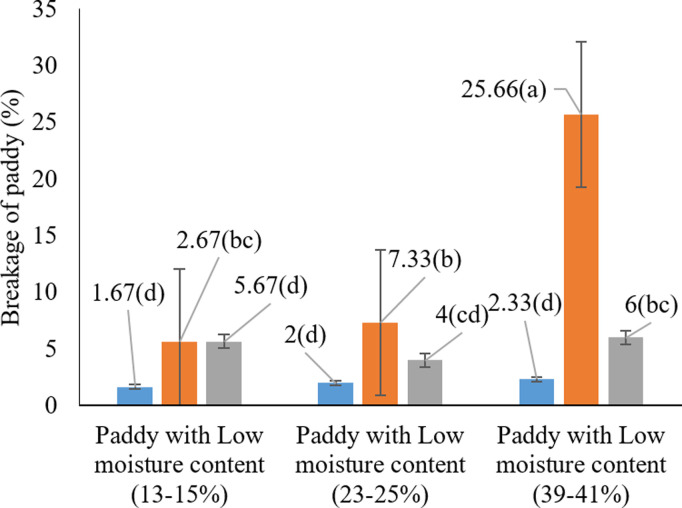
Interaction effects of harvest moisture and harvesting method on paddy cracking * Means with similar letters on each chart do not have a statistically significant difference (LSD).

These differences may stem from the mechanical stress imposed on grains during harvesting, which is typically more intense when using cereal combine harvesters [22]. In line with the findings of Odoom et al. (2021b) [53] who emphasized that drying temperature and grain moisture content directly influence grain cracking it appears that similar effects occur during the harvesting process. As shown in Fig 6, the highest rice grain breakage occurred under the condition of using a cereal combine harvester at high moisture content (39–41%), with an average breakage of 25.66%. This finding indicates that the combination of high moisture levels and cereal combine harvesting inflicts the greatest damage to rice grains. As previously mentioned, high moisture content softens the grains, rendering them more vulnerable to mechanical breakage.

Supporting this observation, prior studies have consistently highlighted the role of grain moisture in determining post-harvest quality. Kianni (2023) [52] emphasized the importance of maintaining optimal moisture levels at harvest, noting that elevated moisture softens grains and leads to increased breakage. Similarly, Ilieva et al. (2025) [44] reported that harvesting rice at moisture contents above 22.8% significantly reduces milling yield and increases grain damage. Also confirmed that operating cereal combines under high-moisture conditions tends to result in greater grain damage.

The results of the present study confirm that a high grain moisture content (39–41%) combined with the use of a cereal combine harvester leads to the highest rate of rice breakage. These findings align with those of Khodabakhshipoor et al. (2011) [54], who investigated the effects of harvesting moisture content, threshing drum speed, and feed rate on rice grain quality using a flow threshing machine. Their study showed that increasing drum speed, across two moisture content levels and feed rates, increased the percentages of broken and husked rice from 0.71% to 1.17% and 0.82% to 1.45%, respectively. Another study conducted in Gilan Province highlighted that economic constraints, operator skill, machinery type, and harvest timing are major contributors to rice grain breakage. The use of unsuitable threshers or outdated combines and the lack of financial resources for equipment upgrades significantly increased grain losses. Financial and educational support were identified as key strategies to reduce such losses [55].

These analyses clearly demonstrate that elevated grain moisture and the use of cereal combine harvesters can significantly exacerbate rice grain breakage. This has critical implications for the quality and efficiency of rice production systems, particularly for future yield optimization.

#### 3.1.3. Effect of harvest moisture and harvesting method on cracked paddy grains.

**Fig 7:**
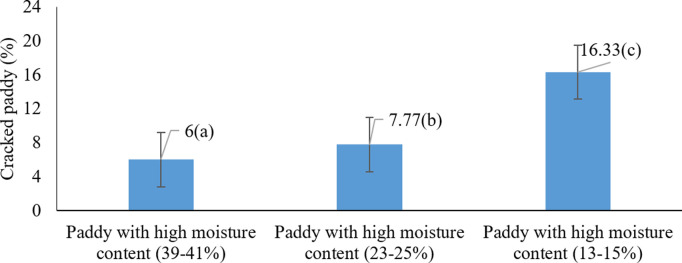
Effects of harvest moisture on cracked paddy grains * Means with similar letters on each chart do not have a statistically significant difference (LSD).

The analysis of variance revealed that the main effects of harvest moisture content and harvesting method, as well as their interaction, were statistically significant on the proportion of cracked paddy grains at the 5% level (Table 1). As illustrated in Fig 7, the highest percentage of cracked rice (16.33%) occurred at the harvest moisture range of 39–41%, while the lowest value (6%) was observed at 13–15% (P < 0.05). These results clearly indicate that increasing moisture content at harvest leads to a higher rate of grain cracking.

These findings are consistent with those of Ilieva et al. (2025), who noted increased grain cracking and reduced resistance at higher moisture levels [44]. Similarly, Alwan-Alsharifi et al. (2011) reported that excessive grain moisture contributes to increased breakage and reduced milling yield, whereas optimal moisture levels enhance grain quality [11]. These results reinforce the importance of carefully managing harvest moisture content to preserve grain integrity and minimize post-harvest losses.

**Fig 8:**
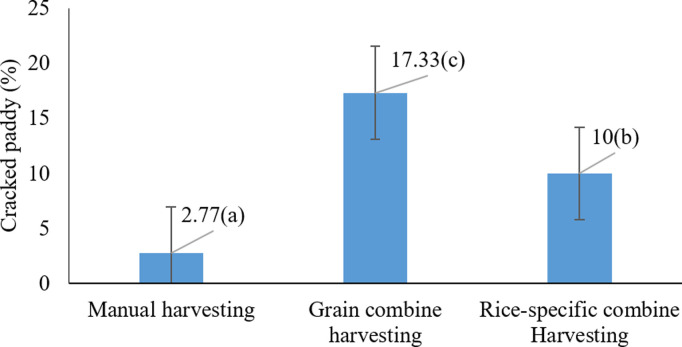
Effects of harvesting method on cracked paddy grains * Means with similar letters on each chart do not have a statistically significant difference (LSD).

According to Fig 8, the harvesting method significantly affected cracked paddy grains, with the cereal combine harvester resulting in the highest rate of cracking (17.33%) (P < 0.05). This observation aligns with the results of Alwan-Alsharifi (2011), who noted increased mechanical stress and damage associated with cereal combine harvesters [11].

In addition, Odoom et al. (2021b) reported that harvesting equipment exerting high mechanical pressure causes grain damage and cracking [47]. Therefore, harvesting method selection has a measurable effect on cracked grain percentage and should be considered a critical factor in rice production.

**Fig 9:**
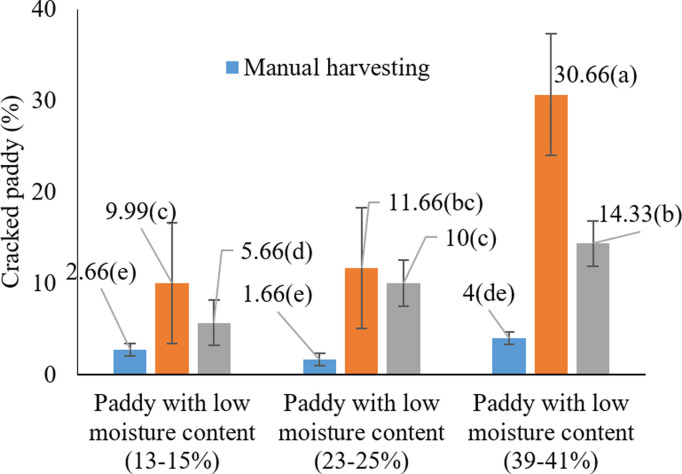
Interaction effects of harvest moisture and harvesting method on cracked paddy grains * Means with similar letters on each chart do not have a statistically significant difference (LSD).

The comparison of the mean interaction effects (Fig 9) showed that the highest level of cracked rice grains was observed with the cereal combine harvester method and high harvest moisture content (P < 0.05). As shown in the chart, the highest percentage of cracked rice grains was observed with the cereal combine harvester method at high harvest moisture (30.66%), while the minimum level was observed with manual harvesting at low harvest moisture (P < 0.05). This indicates that the interaction between harvesting method and moisture level plays a critical role in determining rice grain integrity. These results align with previous studies that have shown that harvest moisture content is a key factor in determining the level of cracking in rice grains.

Various studies emphasize that high moisture content during harvesting can reduce the mechanical strength of the grains and increase the cracking rate. For example, Ilieva et al. (2025) and Alwan-Alsharifi et al. (2017) reported that high moisture content at harvest reduces the mechanical strength of the grains and increases the percentage of cracking [10,44]. In contrast, low moisture content can reduce cracking and improve grain quality, but it may also lead to a decrease in milling yield and processing quality. The present results reinforce these findings by demonstrating a statistically significant increase in cracked grains under high moisture conditions. Furthermore, the findings of this study highlight the importance of adjusting harvesting conditions. Using a cereal combine harvester with high moisture content can cause mechanical damage to the grains and increase product losses. Such results stress the necessity for optimizing both harvest timing and machinery usage to reduce mechanical stress on rice grains. Therefore, it is recommended that in the selection of the harvesting method, along with careful machine settings, the moisture conditions be carefully managed to minimize potential damage to the grains and preserve the quality of the final product.

In the present study, harvest moisture and the harvesting method had a significant impact on the cracking of rice grains. Statistical analysis confirmed that both factors independently and interactively influenced cracking percentage at a significant level (P < 0.05). The drying process is one of the crucial and impactful stages in post-harvest processing of rice, which affects both the quality of its transformation into white rice and the percentage of cracks in the grains. Firouzi and Alizadeh (2014) [47] investigated the effect of harvesting time and harvest moisture content on the cracking and breakage of milled rice and concluded that harvesting 30 days after 50% flowering and a harvest moisture content of 8–9% (dry basis) resulted in the lowest breakage and cracking.

Unlike other cereals, which are typically consumed in flour form, rice is mainly consumed as whole grains. Therefore, the economic value of rice largely depends on its quality after the transformation from paddy to white rice. Milling degree and crack percentage are two important indicators to determine rice quality after milling. Most rice breakage is caused by the presence of fine cracks in the endosperm of the grains. These cracks, often referred to as stress cracks, are created due to a combination of mechanical, moisture, and thermal stresses. One of the key stages for the formation of these cracks is the drying of the paddy, which makes the grains more prone to breakage during further production or processing stages.

Various studies have shown that increased moisture content in paddy at harvest and the post-harvest processing method have a significant impact on rice cracking.

Research indicates that the higher the initial moisture content of the paddy, the higher the likelihood of immature, thin, and chalky grains, which leads to an increase in grain breakage [56]. Moreover, higher drying temperatures and faster moisture reduction result in internal stresses in the grains, consequently increasing cracking [57,58]. The consistency of current results with these findings supports the hypothesis that moisture-induced stress during harvesting and drying is a principal factor contributing to grain cracking. These findings confirm that proper management of harvest conditions and post-harvest processing is essential to reduce losses and preserve the quality of rice grains.

The results of this study also showed that the highest percentage of breakage was observed with the cereal combine harvester method, suggesting that the mechanical forces generated by this method are more severe compared to manual harvesting. This may be due to the increased rotation speed of the combine harvester. These results align with the findings of Alimohammadzadeh et al. (2015) [6], while they contradict the findings of Safari et al. (2014) [42]. Based on the results of this study, it appears that the speed and type of harvesting machinery, particularly the cereal combine harvester, significantly influence the level of cracking in the grains. This highlights the need to re-evaluate combine harvester settings and operational parameters to minimize breakage rates. Therefore, it is recommended that in selecting a harvesting method, attention should not only be given to the moisture content of the grains but also to the rotation speed of the machinery and its proper adjustments to minimize damage to the grains and improve the quality of the final product.

### 3.2. Effect of harvesting moisture and harvesting method on breakage and head rice yield

Table 4 provides the analysis of variance for the effects of paddy moisture content at harvest and harvesting method on Milling degree (MD), Milling recovery (MR), Broken rice percentage (BR %), and the Head rice yield (HRY). The results indicate that the effects of harvest moisture, harvesting method, and their interaction were significant at the 1% probability level for breakage percentage and the percentage of whole rice. However, no significant effect was observed on the milling degree and milling recovery.

**Table 4:** Analysis of variance for the effect of harvest moisture content and harvesting method on paddy traits.

Source of variation	df	Mean squares			
		Milling degree (%)	Milling recovery (%)	Rice breakage (%)	Head rice yield (HRY) (%)
Replication	2	0.5	1.46	9.33	8.33
Harvest moisture content	2	0.93^ns^	0.163^ns^	12.78**	128.78**
Harvesting method	2	2.41^ns^	0.307^ns^	10.41**	100.41**
Harvest moisture content × Harvesting method	4	1.27ns	2.62^ns^	84.72**	80.72**
Error	16	1.44	1.04	2.25	1.25
CV	–	1.5	1.62	7.37	1.88

**: Significant at the 1% level,*: Significant at the 5% level and ^ns^: not significant.

#### 3.2.1. Effects of moisture and harvesting method on breakage and head rice yield (HRY).

**Fig 10:**
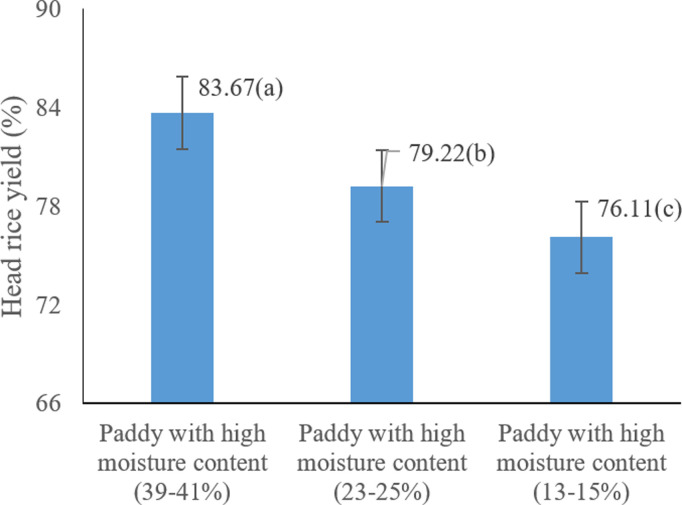
Effects of harvest moisture on the percentage of Head rice yield (HRY) * Means with similar letters on each chart do not have a statistically significant difference (LSD).

The results of the present study clearly demonstrate that lower harvest moisture content significantly improves the percentage of intact head rice yield (HRY). Specifically, rice harvested at 13–15% moisture content yielded the highest average HRY of 83.66% (P < 0.05) (Fig 10**).** These findings are consistent with previous studies, such as Hedayatipour (2005), who reported that elevated grain moisture at harvest adversely affects the degree of milling and the proportion of whole rice [59]. Similarly, Bautista et al. (2009) and Siebenmorgen et al. (2007) concluded that harvesting paddy within an optimal moisture range (18–20%) enhances milling yield and minimizes breakage [17]. Harvesting method also played a critical role in preserving rice quality, with manual harvesting yielding the highest HRY (83.11%). This can be attributed to the minimal mechanical stress imposed during manual operations. In contrast, combine harvesters, due to their rotating and threshing mechanisms, inflict more mechanical damage, leading to a higher proportion of broken grains. These findings align with those of Alwan-Alsharifi et al. (2017), who emphasized the importance of optimal clearance between harvester cylinders and maintaining appropriate moisture levels (10–12%) to reduce grain damage and improve husking efficiency [10]. Moreover, Odoom et al. (2021) noted that precise drying conditions and achieving a final grain moisture of 12–13% are essential to minimize cracking [53].

The statistical analysis confirmed that harvest moisture content, harvesting method, and their interaction had a significant effect (P < 0.01) on the percentage of intact head rice yield (Table 4). These results underscore the importance of harvest timing and technique in reducing post-harvest losses. Elevated grain moisture was consistently associated with increased breakage and lower HRY, reinforcing the negative impact of suboptimal harvest conditions [8,60,61]. Furthermore, modern harvesting technologies, when properly calibrated, show strong potential in reducing mechanical losses. Advanced machinery, operated under optimal conditions, contributes to better grain integrity and improved milling recovery [21]. Aghkhani, & Rohani (2024) further emphasized the critical role of equipment calibration and technological advancement in preserving rice quality during harvest [46].

**Fig 11:**
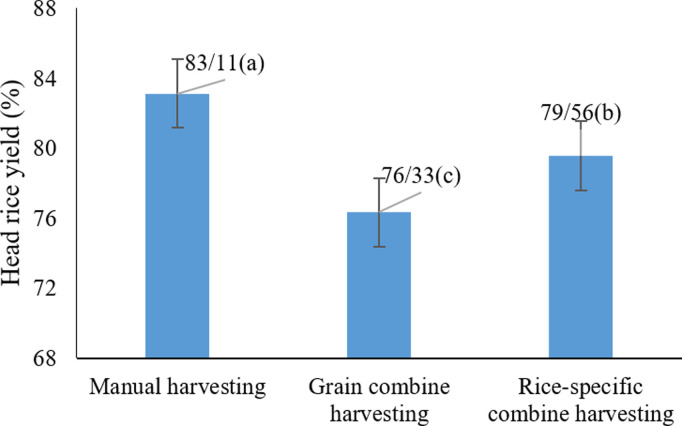
Effects of harvesting method on the percentage of Head rice yield (HRY) * Means with similar letters on each chart do not have a statistically significant difference (LSD).

The analysis of the data (Table 4) revealed that harvest moisture content, harvesting method, and their interaction significantly affected the percentage of intact Head rice yield (HRY) (P < 0.05). Among the evaluated methods, manual harvesting achieved the highest average HRY (83.11%), significantly outperforming mechanized techniques (P < 0.05) (Fig 11). This underscores the critical influence of harvesting method on grain breakage. Specifically, the type of combine harvester played a substantial role in the mechanical stress applied to paddy rice, with general-purpose cereal combines generating higher mechanical forces during threshing, resulting in increased breakage. In contrast, manual harvesting minimized physical damage and better preserved grain integrity. These outcomes align with previous studies [8,60,61], which also emphasized the impact of harvesting techniques on kernel integrity. While mechanized harvesting remains indispensable for large-scale production, optimizing combine harvester settings is essential to reduce mechanical damage and ensure grain quality [22].

**Fig 12:**
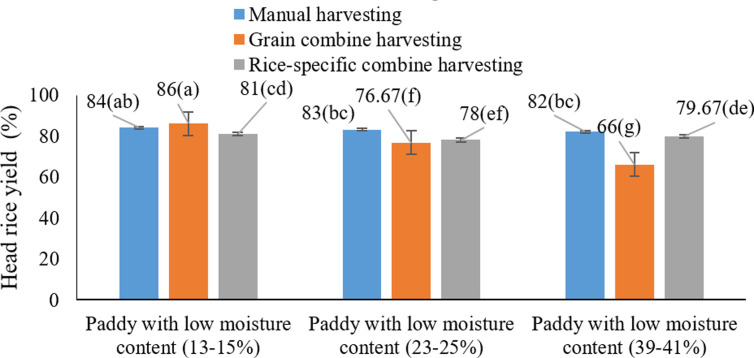
Interaction effects of harvest moisture and harvesting method on the percentage of Head rice yield (HRY) * Means with similar letters on each chart do not have a statistically significant difference (LSD).

The results of the interaction effect analysis (Fig 12) revealed that the combined influence of harvest moisture content and harvesting method had a significant effect on the percentage of intact head rice yield (HRY). The highest HRY values (above 83%) were recorded when rice was harvested at a moisture content of 13–15% using manual harvesting (P < 0.05). Similarly, rice-specific combines maintained relatively high HRY levels under the same moisture conditions. In contrast, cereal combines operating at higher moisture contents (>18%) resulted in the lowest proportions of intact rice. These findings are consistent with the results of the studies that underscored the importance of moisture content and machinery type in determining final grain quality [62].

The data also confirmed that elevated harvest moisture significantly contributes to grain breakage during handling and processing [61]. Furthermore, cereal combines tend to exert greater mechanical stress due to their design, increasing the likelihood of breakage [62]. These outcomes clearly demonstrate that maintaining a harvest moisture content of 13–15%, coupled with either manual or rice-specific mechanized harvesting, optimizes the integrity of white rice.

#### 3.2.2. Effect of harvesting methods on rice breakage.

The analysis of variance results (Table 4) indicated that the effect of harvest moisture content, harvesting method, and their interaction on rice breakage percentage were significant at the 1% level.

**Fig 13:**
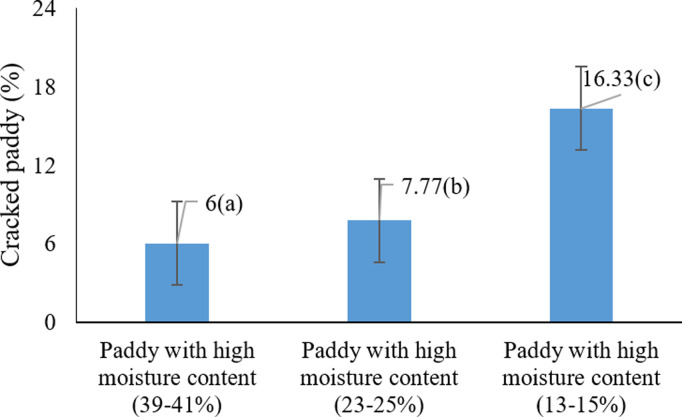
Effect of harvest moisture content on rice breakage * Means with similar letters on each chart do not have a statistically significant difference (LSD).

The comparison of means (Fig 13) revealed that harvest moisture content had a significant impact on rice breakage, with the lowest breakage observed at a harvest moisture content of 13–15%, averaging 16.33% (P < 0.05). Higher harvest moisture content resulted in a statistically significant increase in paddy breakage, with the highest breakage percentage observed at the 19–21% moisture level. A study by Bhattacharya (2011) [63] demonstrated that a gradual reduction in paddy moisture to an optimal range positively impacts the reduction of breakage. The results of this study confirm that proper management of harvest timing, especially harvesting at a moisture content of 13–15%, plays a crucial role in minimizing breakage and improving the final quality of rice [46].

**Fig 14:**
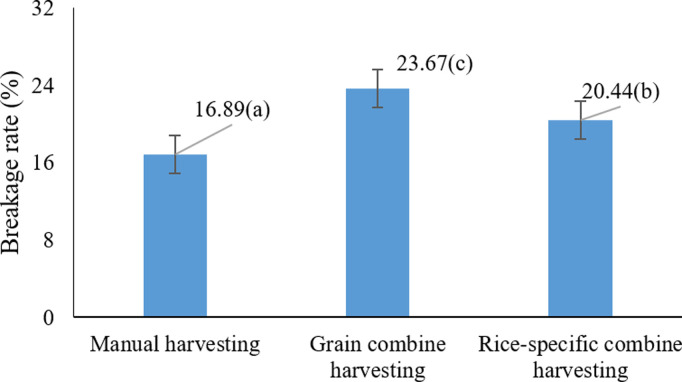
Effects of harvesting method on rice breakage * Means with similar letters on each chart do not have a statistically significant difference (LSD).

The comparison of means (Fig 14) showed that the harvesting method had a significant effect on the percentage of rice breakage (P < 0.05), with the highest average breakage (29.14%) recorded in the cereal combine method, and the lowest (16.88%) in the manual harvesting method.

This significant difference suggests that the mechanical forces involved in cereal combines contribute substantially to grain breakage, whereas the reduced mechanical stress in manual harvesting minimizes breakage levels. Previous studies have also shown that using grain combines, especially when the equipment settings are not optimized for rice, can result in increased grain breakage [63,64].

**Fig 15:**
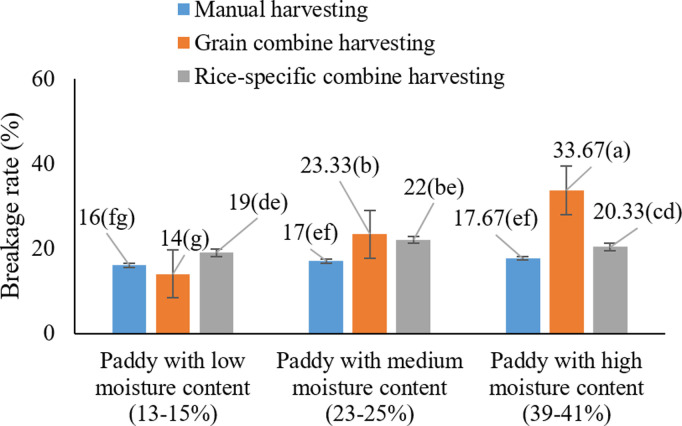
Interaction effects of harvest moisture and harvesting method on rice breakage * Means with similar letters on each chart do not have a statistically significant difference (LSD).

The analysis of variance and subsequent mean comparisons (Fig 15) revealed that the interaction between harvest moisture content and harvesting method had a statistically significant effect on rice breakage. The highest breakage rate (33.66%) was observed when mechanical harvesting using a cereal combine was performed at high grain moisture levels (19–21%), whereas the lowest breakage occurred under manual harvesting at lower moisture levels (13–15%).

This significant interaction highlights the importance of both harvest timing and method in determining final rice quality. Elevated moisture content at harvest increases the susceptibility of rice grains to breakage during milling, as grains with higher moisture are more elastic and more prone to deformation and cracking when subjected to the mechanical stress of husking and whitening rollers [65,66]. Additionally, the type and operation of the harvesting equipment influence grain integrity. Cereal combines, due to their structural design and higher threshing intensity, impose greater mechanical stress on the grains, resulting in increased breakage [64].

The findings of the current study statistically confirm earlier reports indicating that proper calibration of combine settings and harvesting within an optimal moisture window can substantially reduce breakage and preserve rice quality. For example, Bose and Chattopadhyay (1976) reported an optimal harvest moisture range of 22.5–25%. Similarly, Calderwood et al. (1989) observed that delayed harvesting initially increases the proportion of whole milled rice, but beyond a certain point, this proportion starts to decline, indicating a critical threshold for moisture content and harvest timing [60,61].

#### 3.2.3. Effect of harvesting methods on milling recovery (MR).

The data analysis revealed that harvest moisture content, harvesting method, and their interaction had no significant effect on milling recovery (MR) (P > 0.05) (Table 4). All levels of moisture content and harvesting methods yielded statistically similar MR values, indicating no significant differences among the treatments. These results are consistent with previous studies, which demonstrated that under standard milling conditions, variations in harvest moisture and harvesting techniques do not significantly influence milling yield [63,66]. Although no notable effects were observed in this study, earlier research has highlighted that other factors such as rice variety, drying method, and equipment calibration—may play a more prominent role in determining milling performance [64,65].

#### 3.2.4. Evaluation of the effect of harvesting methods on milling degree (MD).

The analysis of variance showed no significant effects (P > 0.05) of harvest moisture content, harvesting method, or their interaction on the milling degree (MD), as reflected in Table 4. Treatment comparisons revealed statistically similar MD values across all levels of moisture content and harvesting techniques. These findings are supported by previous literature, which suggests that head rice yield and milling degree are generally unaffected by harvest method or timing under standard field conditions, and are more influenced by post-harvest handling processes such as drying and storage [66].

## Conclusion

This study examined the effects of different harvesting methods and grain moisture contents on rice losses, breakage, and grain quality in the Champa variety under field conditions. The results confirmed the inefficiency of traditional practices, with manual harvesting leading to the highest paddy losses (7.83%), while rice-specific combine harvesting demonstrated the lowest losses (3.38%). Grain moisture played a critical role, as high moisture levels (39–41%) significantly increased both paddy losses (5.83%) and grain breakage (11.33%), whereas harvesting at lower moisture levels (13–15%) minimized both.

The interaction between harvesting method and grain moisture revealed that grain combines caused higher losses at low moisture levels due to increased brittleness and field-induced stress, while elevated moisture levels led to higher breakage rates across all methods. Moreover, the highest incidence of broken grains occurred at moderate moisture levels (23–25%) under grain combine use, whereas the maximum head rice yield (HRY) (86%) was achieved through manual harvesting at 13–15% moisture.

These results are in agreement with previous findings, reinforcing the substantial impact of both harvesting method and timing on post-harvest outcomes. The study emphasizes the importance of adopting data-driven harvesting strategies, including weather-based scheduling, operator training, and investments in drying infrastructure.

In regions vulnerable to labor shortages or climate risks, such adaptive strategies can improve grain quality and economic returns. Future research should focus on integrating real-time moisture sensing technologies, precision combine calibration, and exploring hybrid harvesting systems to balance efficiency and grain integrity in high-value rice production.

Ultimately, aligning harvesting decisions with grain physiology and environmental conditions is essential to ensuring sustainable, high-quality rice production in the face of evolving agronomic and climatic challenges.

## Supporting information

S1 Data. (XLSX)
